# Unpredictable Nature of Tolvaptan in Treatment of Hypervolemic Hyponatremia: Case Review on Role of Vaptans

**DOI:** 10.1155/2014/807054

**Published:** 2014-01-08

**Authors:** Ishan Malhotra, Shilpa Gopinath, Kalyana C. Janga, Sheldon Greenberg, Shree K. Sharma, Regina Tarkovsky

**Affiliations:** ^1^Department of Internal Medicine, Maimonides Medical Center, Brooklyn, NY, USA; ^2^Department of Nephrology, Maimonides Medical Center, 953 49th Street, Brooklyn, NY 11219, USA

## Abstract

Hyponatremia is one of the most commonly encountered electrolyte abnormalities occurring in up to 22% of hospitalized patients. Hyponatremia usually reflects excess water retention relative to sodium rather than sodium deficiency. Volume status and serum osmolality are essential to determine etiology. Treatment depends on several factors, including the cause, overall volume status of the patient, severity of hyponatremic symptoms, and duration of hyponatremia at presentation. Vasopressin antagonists like tolvaptan seem promising for the treatment of euvolemic and hypervolemic hyponatremia in heart failure. Low sodium concentrations cause cerebral edema, but the overly rapid sodium correction can also lead to iatrogenic cerebral osmotic demyelination syndrome. Demyelination may occur days after sodium correction or initial neurologic recovery from hyponatremia. The following case report analyzes the role of vasopressin antagonists in the treatment of hyponatremia and the need for daily dosing of tolvaptan and the monitoring of serum sodium levels to avoid rapid overcorrection which can result in osmotic demyelination syndrome (ODS).

## 1. Introduction

Hyponatremia which is defined as serum sodium levels less than 135 mmol/L is often encountered in patients with heart failure. Patients with heart failure develop hyponatremia due to the activation of neurohormonal system leading to decrease in sodium levels. Treatment options for hyponatremia in heart failure, such as water restriction or the use of hypertonic saline with loop diuretics, have limited efficacy. AVP-receptor antagonists increase sodium levels effectively and their use has proven to be effective in correcting sodium levels and improving the outcome of these patients. However, their safety in terms of overcorrecting sodium levels with daily doses of 15–60 mg of tolvaptan is still debatable. Rapid correction of sodium levels in chronic hyponatremia patients has been shown to cause hypernatremia and osmotic demyelination syndrome (ODS) with grave consequences.

We report a case of a 51-year-old male who was admitted with chronic hypervolemic hyponatremia. He developed acute hypernatremia and osmotic demyelination syndrome due to administration of tolvaptan and diuretics. We raise the question of dosing of vasopressin antagonists only after checking daily sodium levels and monitoring urine output.

## 2. Case Presentation

**Figure 1:**
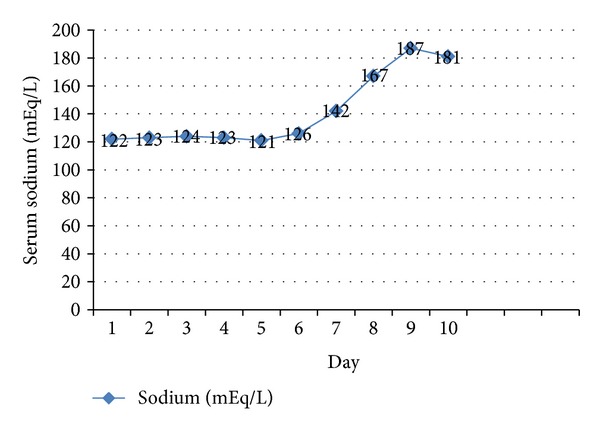
Effect of tolvaptan on serum sodium levels.

**Figure 2:**
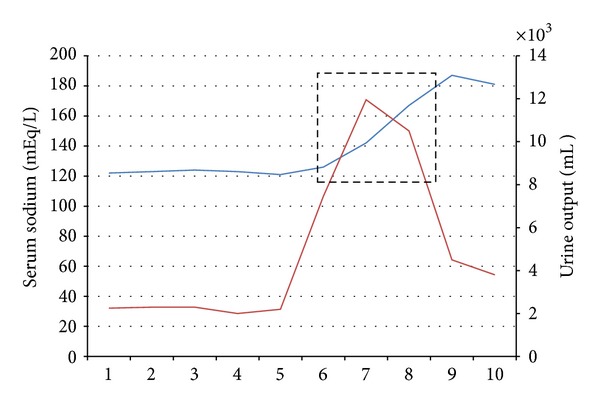
Effect of tolvaptan on serum sodium and urine output. The dotted rectangle shows the exposure to Tolvaptan from day 6 to day 9.

A 51-year-old male with past medical history of coronary artery disease and peripheral vascular disease presented to the hospital with progressive shortness of breath and bilateral pedal edema. On admission the patient had a B type natriuretic peptide level of 3458, sodium of 122 mmol/L, potassium of 5.2 mmol/L, and blood urea nitrogen/creatinine ratio of 39/1.5. Echocardiogram showed global hypokinesis with an ejection fraction of 10–15%. (Relevant lab values are shown in Table 1.) Due to hyponatremia aggressive diuresis was done and as there was no rise in serum sodium levels, tolvaptan 15 mg was started on hospital day 6. The patient showed improvement after receiving the first dose of tolvaptan and on hospital day 7 his serum sodium was 126 mmol/L (see Figure 1). On day 8 he had a rapid increase in his serum sodium level from 126 mmol/L to 142 mmol/L after he received the second dose of tolvaptan. His serum sodium levels further increased from 159 mmol/L to 167 mmol/L on day 8. At this point, tolvaptan was stopped. (The effects of tolvaptan on serum sodium levels and urine output have been shown in Figure 2.) The patient developed signs of osmotic demyelination syndrome which failed to resolve after rapid correction with hypotonic fluids and desmopressin and was transferred to the medical intensive care unit for further management of hypernatremia.

## 3. Discussion

Hyponatremia is defined as a plasma Na^+^ concentration <135 mmol/L. This disorder is always almost the result of an increase in circulating AVP and/or increased renal sensitivity to AVP, combined with an intake of free water. Hyponatremia usually reflects the retention of excessive water relative to sodium rather than sodium deficiency alone [1, 2]. It is subdivided* diagnostically* into 3 groups depending on the clinical history and volume status: hypovolemic, euvolemic, and hypervolemic. Euvolemic hyponatremia has a broad differential diagnosis. Most processes are mediated directly or indirectly through ADH, including hypothyroidism, adrenal insufficiency, medications, and the syndrome of inappropriate ADH (SIADH). Hypervolemic hyponatremia occurs in the edematous states of cirrhosis, heart failure, nephrotic syndrome, and advanced kidney disease [3]. In cirrhosis and heart failure, effective circulating volume is decreased due to peripheral vasodilation or decreased cardiac output. Increased renin-angiotensin-aldosterone system activity and ADH secretion result in water retention. Euvolemic patients may respond to free water restriction alone. Hypervolemic patients may require loop diuretics or dialysis, or both, to correct increased total body water and sodium. Although loop diuretics are an essential component of therapy for acute decompensated heart failure, clinical trials have found no significant differences in either patients' global assessment of symptoms or the change in the creatinine level from baseline to 72 hours when diuretic therapy was administered by means of boluses as compared with continuous infusion or with a low-dose strategy as compared with a high-dose strategy [4]. Vasopressin antagonists like tolvaptan given orally have revolutionized the treatment of both euvolemic and hypovolemic hyponatremia especially in heart failure [3].

Arginine vasopressin (AVP) is a neuropeptide hormone synthesized in the nuclei of the hypothalamus in neuronal cell bodies and released from the posterior pituitary into the bloodstream. AVP has two different receptor subtypes: V1 and V2 [5]. V1 receptors are further subdivided into V1a and V1b. V1a receptors are found in vascular smooth muscle and cardiac myocytes causing vasoconstriction and hypertrophy, as well as in platelets and hepatocytes regulating platelet aggregation and glycogen metabolism [6, 7]. V1b receptors are found in the anterior pituitary gland and are associated with adrenocorticotropic hormone and b-endorphin release [8]. V2 receptors are found on the renal collecting ducts, cause free-water reabsorption leading to increased water retention, and are mainly linked to the development of hyponatremia in heart failure patients. In the 1990s, a number of nonpeptide vasopressin antagonists were discovered. Tolvaptan (OPC-41061) is one of the potent, highly selective, and orally effective nonpeptide antagonists. Vaptans are nonpeptide competitive inhibitors of the V2 receptor located on the basolateral membrane of the collecting ducts principal cells. They bind to the V2 receptor, preventing the hormone's downstream signaling pathway—the generation of intracellular cAMP and the expression and insertion of aquaporin-2 on the apical membrane. This inhibits water reabsorption and results in the excretion of markedly dilute urine (aquaresis) [9]. Tolvaptan is available in strengths of 15 mg and 30 mg [10]. Approximately 40% of the drug is absorbed after oral administration. The onset of effect is two to four hours after a dose is taken, and peak effects occur four to eight hours after administration. The volume of distribution is approximately 3 L/kg, and it is increased in patients with moderate-to-severe hepatic impairment and heart failure; however, this does not affect the patient's response to the drug. Renal impairment is not known to affect the distribution of tolvaptan [10]. The terminal-phase half-life is 12 hours; however, increased serum sodium concentrations persist at 24 hours after dose despite a return to baseline free water excretion.

Overly rapid correction of hyponatremia (e.g., >12 mEq/L/24 hours) with tolvaptan can cause osmotic demyelination resulting in dysarthria, mutism, dysphagia, lethargy, affective changes, spastic quadriparesis, seizures, coma, and death. In susceptible patients, including those with severe malnutrition, alcoholism or advanced liver disease, slower rates of correction may be advisable. Osmotic demyelination syndrome is a serious neurologic disorder which in the well-adjusted intracellular environment becomes relatively hypotonic, with rapid or complete restoration of extracellular osmolality. Water then flows from the intracellular to the extracellular compartment, causing cell volume collapse. In the CNS this can cause a demyelinating lesion [11]. ODS is suggested to develop by the intracellular dehydration of oligodendrocytes in the pons and other susceptible areas by hyperosmotic stimulus, which induces oligodendrocyte apoptosis and demyelination [10]. Slow correction of serum Na (less than 12 mmol/L/day) is recommended to prevent the emergence of this syndrome [10]. There is no standard therapy once ODS sets in. Fluid should not be restricted in patients with hyponatremia who are started on AVP-receptor antagonists and serum sodium concentration should be monitored every 6–8 h in order to avoid rapid correction of sodium levels [12]. Rapid reinduction of hyponatremia with hypotonic fluids like 5% dextrose water and half normal saline along with desmopressin has been shown to improve mortality and even reverse the signs and symptoms of ODS and is currently the favored strategy to treat patients with ODS due to hypernatremia [13–16].

## 4. Conclusion

The present case tells us that severe chronic hyponatremia must be managed with extreme care especially in patients with chronic debilitating illness due to the unpredictable nature of tolvaptan to raise serum sodium levels. In most hospital settings, warfarin dosing happens after checking daily prothrombin time. Similarly tolvaptan should be administered after checking serum sodium levels for that day and it should not be started as a standing order while initiating treatment.

## Figures and Tables

**Table 1:** Trend of pertinent clinical and laboratory data.

Day	Sodium (mEq/L)	Urine output (mL)	Tolvaptan (mg)
1	122	2250	—
2	123	2300	—
3	124	2300	—
4	123	2000	—
5	121	2200	—
6	126	7460	15
7	142	11950	15
8	167	10500	30
9	187	4500	30
10	181	3800	—
